# Decontamination of Titanium Surface Using Different Methods: An In Vitro Study

**DOI:** 10.3390/ma13102287

**Published:** 2020-05-15

**Authors:** Sifan Yan, Min Li, Satoshi Komasa, Akinori Agariguchi, Yuanyuan Yang, Yuhao Zeng, Seiji Takao, Honghao Zhang, Yuichiro Tashiro, Tetsuji Kusumoto, Yasuyuki Kobayashi, Liji Chen, Kosuke Kashiwagi, Naoyuki Matsumoto, Joji Okazaki, Takayoshi Kawazoe

**Affiliations:** 1 Department of Removable Prosthodontics and Occlusion, Osaka Dental University, 8-1 Kuzuha-hanazono-cho, Hirakata, Osaka 573-1121, Japan; yan-z@cc.osaka-dent.ac.jp (S.Y.); liminmin0529@gmai.com (M.L.); komasa-s@cc.osaka-dent.ac.jp (S.K.); akinori@agariguchi.com (A.A.); yang-y@cc.osaka-dent.ac.jp (Y.Y.); zeng-y@cc.osaka-dent.ac.jp (Y.Z.); takao-s@cc.osaka-dent.ac.jp (S.T.); zhang-h@cc.osaka-dent.ac.jp (H.Z.); tashiro@cc.osaka-dent.ac.jp (Y.T.); 2 Department of Oral Health Engineering, Faculty of Health Sciences, Osaka Dental University, Osaka 573-1121, Japan; kusumoto@cc.osaka-dent.ac.jp; 3 Osaka Research Institute of Industrial Science and Technology Morinomiya Center, 1-6-50, Morinomiya, Joto-ku, Osaka-shi 536-8553, Japan; kobaya@omtri.or.jp; 4 Department of Orthodntics, Osaka Dental University, 8-1 Kuzuha-hanazono-cho, Hirakata, Osaka 573-1121, Japan; chen-li@cc.osaka-dent.ac.jp (L.C.); naoyuki@cc.osaka-dent.ac.jp (N.M.); 5 Department of Fixed Prosthodontics, Osaka Dental University, 8-1 Kuzuha-hanazono-cho, Hirakata, Osaka 573-1121, Japan; kashiwagi-d.c@mopera.net (K.K.); kawaoze@cc.osaka-dent.ac.jp (T.K.)

**Keywords:** Ti implant, hydrocarbon decontamination, Finevo cleaning system, ultraviolet treatment, plasma

## Abstract

Contamination of implants is inevitable during different steps of production as well as during the clinical use. We devised a new implant cleaning strategy to restore the bioactivities on dental implant surfaces. We evaluated the efficiency of the Finevo cleaning system, and Ultraviolet and Plasma treatments to decontaminate hydrocarbon-contaminated titanium disks. The surfaces of the contaminated titanium disks cleaned using the Finevo cleaning system were similar to those of the uncontaminated titanium disks in scanning electron microscopy and X-ray photoelectron spectroscopy analysis, but no obvious change in the roughness was observed in the scanning probe microscopy analysis. The rat bone marrow mesenchymal stem cells (rBMMSCs) cultured on the treated titanium disks attached to and covered the surfaces of disks cleaned with the Finevo cleaning system. The alkaline phosphatase activity, calcium deposition, and osteogenesis-related gene expression in rBMMSCs on disks cleaned using the Finevo cleaning system were higher compared to those in the ultraviolet and plasma treatments, displaying better cell functionality. Thus, the Finevo cleaning system can enhance the attachment, differentiation, and mineralization of rBMMSCs on treated titanium disk surfaces. This research provides a new strategy for cleaning the surface of contaminated titanium dental implants and for restoration of their biological functions.

## 1. Introduction

Owing to its good biocompatibility and mechanical processing properties, titanium has been widely used for dental implants [1]. A dental implant is considered to be an appropriate option for replacement of missing teeth [2]; however, the biomechanical and/or biological imbalances between the implant surface and surrounding tissues cause the failure of these implants [3,4]. Biological failure is mostly associated with the accumulation of microbial plaque and bacterial infections [5,6,7], which cause inflammation in the tissues (both soft and hard) surrounding the implant. The inflammatory lesions in peri-implant mucositis affecting the tissues surrounding an osseointegrated implant—in the condition referred to as peri-implantitis—result in the loss of the supporting bone [8]. The etiology of peri-implantitis includes many factors, such as the implant design, poor oral hygiene, degree of roughness, and bacterial contamination of the recipient site [9]. These factors affect the contact between the implant and bone tissue, and it is unable to realize osseointegration [10] wherein there is a direct contact of the fiber-free connective tissue interface layer at the implant–bone interface. Previous studies have shown that carbon deposition on the titanium surface decreases the differentiation of osteoblasts but increases the fibroblasts in the soft tissues [11]. It has also been suggested in some studies that the removal of carbon may be an important step in promoting the bioactivity and osseointegration around titanium implants while preventing soft tissue intervention [12]. It has been reported that titanium implants for clinical and experimental use are contaminated with hydrocarbons [13,14,15]. For clinical use and in the implant production process, the contamination of implants may be inevitable during different procedures, such as cleaning, disinfection, packaging, distribution, and storage. Hence, attention should be paid as to how hydrocarbon decontamination can be done and clearness and roughness of the implant surface can be ensured for arresting peri-implantitis and achieving osseointegration. Many approaches for decontamination of the implant surface have been proposed in the literature. For example, it has been reported that ultraviolet (UV) treatment can increase the bioactivity of the titanium surface and can simultaneously remove the hydrocarbon through two possible mechanisms: the induced photocatalytic activity of TiO_2_ and direct decomposition of the hydrocarbons [15,16,17] by UV treatment at a peak wavelength of 250 nm [18]. Additionally, plasma offers a safe and effective method for cleaning and disinfection. Few studies [19,20] have focused on eliminating the organic substances. The mechanisms of plasma etching at atmospheric pressure are not yet fully clear, but the main mechanism might be based on the process of oxidation [21]. Ultrasonic cleaning has been proven to be a suitable cleaning method, and it is recommended that before the first use of a dental implant it should be cleaned ultrasonically [22].

To date, there is no proven method that can be considered a gold standard for decontamination of implant surfaces. Recently, we discovered a new disinfecting agent called Finevo that can reduce debris and can enhance the implant healing time [23,24]. We believe that the ultrasonic cleaning process could be an ideal decontamination procedure for cleaning the implant surface before clinical use. In the present study, we soaked titanium disks in tallow, which is an organic compound derived from animal fat, to construct a contamination model. There are few studies on organic matter pollution like human sebum or machine oil of dental implants. Besides, tallow is originally a colorless substance, but in order to facilitate the observation of contamination in the course of the experiment, we made it red. Additionally we used three approaches for cleaning, namely the Finevo cleaning system, Ultraviolet treatment, and Plasma treatment. The surface properties were analyzed by scanning electron microscopy (SEM) [25], scanning probe microscopy (SPM), and X-ray photoelectron spectroscopy (XPS). Furthermore, cell morphology, viability, functionality, and features of osteogenesis were examined by culturing the rat bone marrow mesenchymal stem cells (rBMMSCs) on the treated titanium disks, and the decontamination efficiency of three cleaning methods was evaluated to find a new implant cleaning method suitable for clinical use. The results of the present study should provide a better cleaning method for contaminated implants for clinical applications and for use in production processes to restore the bioactivity.

## 2. Materials and Methods 

### 2.1. Sample Preparation

The study was performed on titanium (Ti) disks (diameter 10 mm, thickness 1 mm). The titanium disks prepared using a machine (Engineering Test Service; Osaka, Japan) were subsequently polished sequentially using different grades of abrasive paper (Waterproof Paper^®^ No. 800 and 1000; Riken Corundum Co. Ltd., Saitama, Japan). Thereafter, the disks were rinsed in an ultrasonic machine with acetone, ethanol, and deionized water, in this order (each rinse was for 10 min), and dried overnight at room temperature. All the Ti disks were sterilized using dry heat at 170 °C for 1 h. 

**Figure 1:**
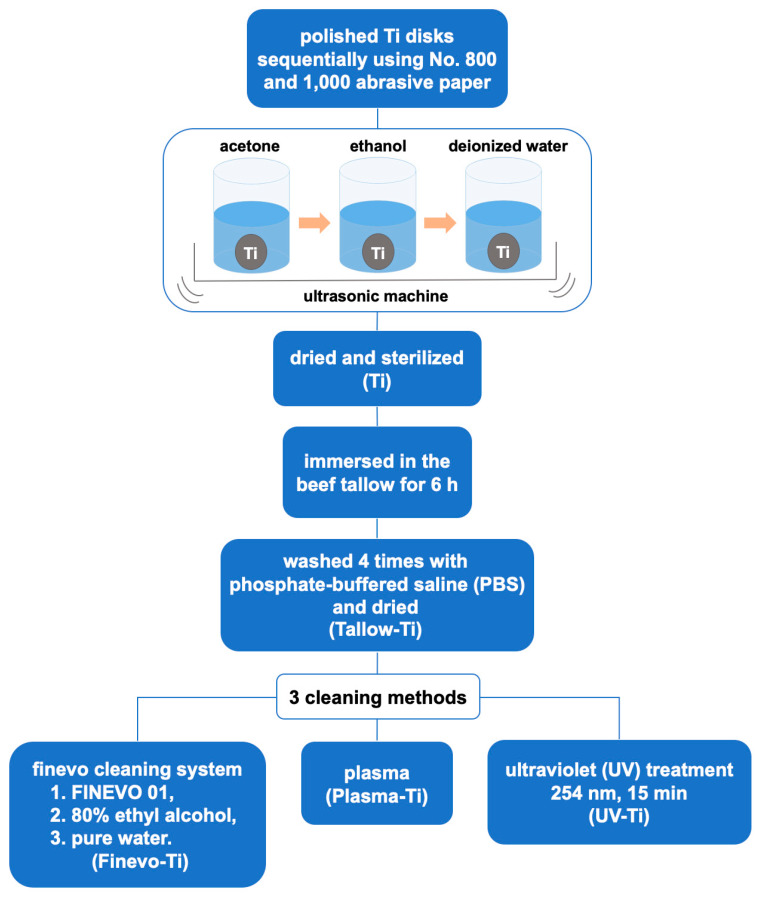
Illustration of contamination construction and three cleaning methods.

To prove whether the three methods can effectively remove the organic contamination on the Ti surface, a contaminated model was constructed using the following procedure: Beef tallow was sterilized by autoclaving at 115 °C for 5 min; the samples were immersed in the beef tallow for 6 h, and washed four times with phosphate-buffered saline (PBS). All the contaminated samples (Tallow-Ti) were dried on a clean bench. The contaminated samples were cleaned using the following three methods: 1. Finevo-Ti method: Samples were cleaned three times in an ultrasonic machine at 30 °C for 5 min each time. The first bath contained an antibacterial cleaning solution (FINEVO 01; Sirius Ceramics, Frankfurt, Germany), the second bath contained 80% ethyl alcohol, and the third bath contained medically pure water (Aqua Dest). 2. Plasma-Ti method: Samples were treated thrice with plasma (Piezobrush^®^ PZ2 plasma handheld device; RELYON Plasma GmbH, Regensburg, Germany) under atmospheric pressure, each time for 10 s. 3. UV-Ti: Samples were treated with ultraviolet light (wavelength 254 nm, strength 100 mW/cm^2^) for 15 min using an ultraviolet irradiation machine (HL-2000 HybriLinker; Funakoshi, Tokyo, Japan). All the treated samples were dried on a clean bench. (Figure 1)

### 2.2. Surface Characterization

The surface morphology of Ti disks, contaminated Tallow-Ti disks, and Ti disks cleaned using the three methods (Finevo-Ti, UV-Ti, and Plasma-Ti) was examined by scanning electron microscopy (SEM; S-4800; Shimadzu, Kyoto, Japan) at a 5 kV accelerating voltage. The mean average surface roughness (Ra) and surface topography were assessed using a scanning probe microscope (SPM; SPM-9600; Shimadzu, Kyoto, Japan). The range of analysis was 125 μm *×* 125 μm. To compare the elemental composition of the Ti surface before and after cleaning using the three methods, the samples were analyzed using X-ray photoelectron spectroscopy (XPS; PHI X-tool; ULVAC-PHI, Kanagawa, Japan) equipped with a monochromatic X-ray source (Al Kα anode) operating at 15 kV and 13 W. The diameter of the analysis point was about 55 μm, and the angle between the electronic analyzer and the sample surface was 45 degrees. 

### 2.3. Cell Culture

Rat bone marrow mesenchymal stem cells (rBMMSCs) were obtained from the femur of 8-week-old Sprague-Dawley rats (Shimizu Laboratory Supplies Co., Kyoto, Japan) and cultured using Eagle’s minimum essential medium (E-MEM) containing 10% fetal bovine serum (FBS) and an antibiotic-antimycotic solution (all from Nacalai Tesque, Kyoto, Japan) in 75-cm^2^ flasks as described previously [26]. The in vitro experiments were carried out using cells in the third passage. The cells were digested with a solution containing 0.5 g/L trypsin and 0.53 mmol/L EDTA (Nacalai Tesque). The cell suspension was centrifuged and the cell pellet was resuspended, and added to the Tallow-Ti, Finevo-Ti, Plasma-Ti, and UV-Ti samples placed in a 24-well plate at a density of 4 × 10^4^ cells/well. The culture medium was changed every 3 d. This study was performed in accordance with the Guidelines for Animal Experimentation at Osaka Dental University (Approval No. 19-06002).

### 2.4. Cell Morphology

After incubation for 24 h, the samples were washed with PBS thrice, and the cells were then fixed with 1 mL 4% paraformaldehyde solution and incubated for 20 min at room temperature. Subsequently, the samples were washed with PBS three times and 0.2% (v/v) Triton X-100 was added to permeabilize the cells. After shaking for 30 s and further incubating for 30 min, the samples were treated with Blocking One reagent (Nacalai Tesque Inc., Kyoto, Japan) for 30 min at room temperature and stained with phalloidin and 4′,6-diamidino-2-phenylindole (DAPI) at 37 °C in the dark for 1 h. The stained samples were washed with PBS three times and subsequently f-actin and cell nuclei were visualized with a confocal laser-scanning microscope (LSM700; Carl Zeiss AG, Wetzlar, Germany).

### 2.5. Cell Adhesion 

CellTiter-Blue^®^ Cell Viability Assay (Promega Corporation, Madison, WI, USA) was used to evaluate the adhesion of cells to the samples of Tallow-Ti and Finevo-Ti at 3 and 24 h, according to the manufacturer’s protocol. The samples that had been incubated for 3 and 24 h were washed with PBS twice and 300 μL of diluted Cell Titer-Blue^®^ reagent (50 μL Cell Titer-Blue^®^ reagent in 250 μL PBS) was added. After 1 h of culture at 37 °C under an atmosphere of 5% CO_2_, 100 μL of the reagent was added in each well of a 96-well plate. The fluorescence was analyzed with a microplate reader (SpectraMax M5; Molecular Devices, San Jose, CA, USA) at excitation and emission wavelengths of 560 and 590 nm, respectively.

### 2.6. Alkaline Phosphatase (ALP) Assay

After 1 week of culture, the cells were placed in a differentiation-inducing medium, which consisted of α-MEM (Nacalai Tesque Inc.), containing 10% FBS, antibiotic–antimycotic mix, and the following osteogenic supplements: 10 mM β-glycerophosphate (Wako Pure Chemical Industries, Osaka, Japan), ascorbic acid (Nacalai Tesque Inc.), and 10 nM dexamethasone (Nacalai Tesque Inc.). This medium was changed every 3 days. At 7 or 14 days of culture, the samples were washed with PBS and 300 μL 0.2% Triton X-100 was added to lyse the cells. The lysates were transferred to microcentrifuge tubes. The Alkaline Phosphatase Yellow (pNPP) Liquid Substrate System for ELISA kit (Sigma-Aldrich, St Louis, MO, USA) was used to detect the ALP activity according to the manufacturer’s protocol. The reaction was terminated by adding 50 μL of 3 M NaOH to 200 μL of the reaction mixture. The production of *p*-nitrophenol (pNP) in the reaction was measured at an optical density of 405 nm using a 96-well microplate reader (SpectraMax M5; Molecular Devices, San Jose, CA, USA). The DNA content was determined with the PicoGreen dsDNA assay kit (Thermo Fisher Scientific, Waltham, MA, USA), following the manufacturer’s protocol. The content of ALP was normalized to the DNA content in the corresponding cell lysates.

### 2.7. Quantification of Calcium Deposition in the Extracellular Matrix

When the samples were cultured with differentiation-inducing medium for 21 and 28 d as described in the “Alkaline phosphatase (ALP) activity”, the calcium deposition in the extracellular matrix was dissolved with 10% formic acid and collected. A Calcium E-Test Kit (Wako Pure Chemical Industries Ltd., Osaka, Japan) was utilized to quantify the amount of calcium. Fifty microliters of the collected medium was mixed with 1 mL calcium emission test reagent and 2 mL kit buffer. Then the reaction products were measured using a 96-well microplate (SpectraMax M5; Molecular Devices) reader at 610 nm. The concentration of calcium ions was calculated according to the absorbance of the relative standard curve.

### 2.8. Analysis of the Expression Levels of Osteogenesis-Related Genes

A real-time TaqMan reverse transcriptase polymerase chain reaction (RT-PCR) assay (Life Technologies, Carlsbad, CA, USA) was used for analyzing the expression levels of osteogenesis-related genes, as previously described [27]. After 3, 7, 14, and 21 d of culture on the samples, total RNA was extracted from rBMMSCs using the RNeasy Mini Kit (Qiagen, Venlo, The Netherlands). Equal amounts (10 μL) of RNA samples were reverse transcribed into cDNA using the PrimeScript RT kit (TaKaRa Bio, Shiga, Japan). The expression levels of *ALP* and runt-related transcription factor 2 (*Runx2*) were quantitatively analyzed at 3 and 7 d, and of bone morphogenetic protein 2 (*BMP-2*) and bone γ-carboxyglutamate (gla) protein (*Bglap*) were analyzed at 14 and 21 d using StepOneTM Plus RT-PCR System (Life Technologies, Carlsbad, CA, USA). The relative gene expression in each group was normalized to that of the housekeeping gene, glyceraldehyde 3-phosphate dehydrogenase (GAPDH), using the ΔΔCt method.

### 2.9. Statistical Analysis

The surface analysis and in vitro experiments were conducted in triplicate. All the quantitative results are expressed as means ± standard deviation. Data were analyzed by one-way analysis of variance (ANOVA) and Bonferroni’s post hoc test using the SPSS 20.0 software (IBM Corporation, Armonk, NY, USA). A value of *p* < 0.05 was considered to be significant, and *p* < 0.01 was considered to be highly significant. 

## 3. Results

### 3.1. Surface Characterization

**Figure 2:**
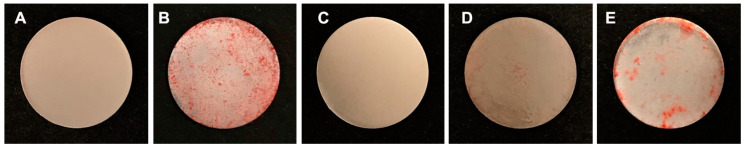
Gross appearance of titanium (Ti) (**A**), Tallow-Ti (**B**), Finevo-Ti (**C**), Plasma-Ti (**D**), and UV-Ti (**E**). Dirt on the surface of the sample dipped in tallow was removed by using Finevo.

**Figure 3:**
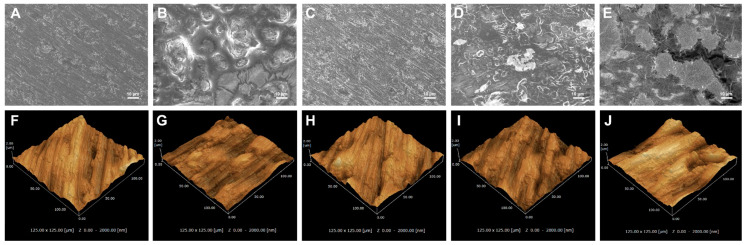
Characterization of the surfaces of the samples. (**A**–**E**) Scanning electron micrograph of Ti, Tallow-Ti, Finevo-Ti, Plasma-Ti, and UV-Ti (magnification = 1000×), scale bar = 10 μm. (**F**–**J**) Scanning probe microscopic images of Ti, Tallow-Ti, Finevo-Ti, Plasma-Ti, and UV-Ti.

An examination of the sample surface (Figure 2) revealed that tallow, the red substance on Ti disks, was attached to Tallow-Ti, Plasma-Ti, and UV-Ti. It was evident from the SEM analysis that Ti and Tallow-Ti surfaces had totally different surface morphologies (Figure 3A,B). The morphological structure of Ti could not be seen in the Tallow-Ti sample. This confirms that beef tallow was completely attached to the surface of Ti. The surface morphology of Finevo-Ti was similar to that of Ti (Figure 3C). The surface characteristics similar to Ti were rarely observed on the surface of Plasma-Ti (Figure 3D), and most of the structures resembled the morphology of Tallow-Ti. However, the Tallow-Ti surface was different from that of UV-Ti, (Figure 3E) and a morphology similar to that of the Ti surface was not seen. The nanotopography of Ti, Tallow-Ti, Finevo-Ti, Plasma-Ti, and UV-Ti surfaces is shown in Figure 3F–J. No significant differences in SPM results were observed among the groups.

**Figure 4:**
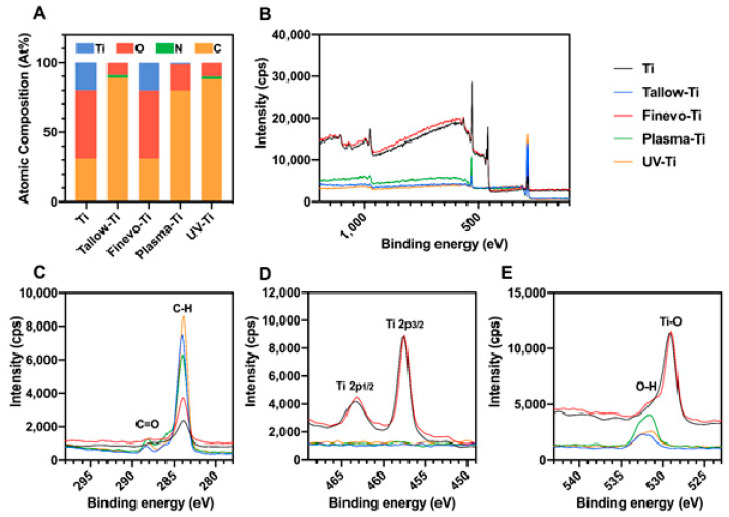
High-resolution chemical analysis of sample surfaces by X-ray photoelectron spectroscopy. (**A**) Atomic percentage of each element on the sample surface. (**B**) High-resolution XPS spectra of sample surfaces. (**C**) C 1s spectra. (**D**) Ti 2p spectra. (**E**) O 1s spectra.

The results of XPS analysis are shown in Figure 3. Compared to the XPS spectrum of Ti, there were not Ti2p and O1s (530.0 eV) corresponding to Ti-O in Tallow-Ti (Figure 4B,D,E). The peak of C1s on Tallow-Ti, at a binding energy of 285.0 eV with a dominant peak corresponding to the hydrocarbon (−CH), was obviously higher than that on Ti (Figure 4C). The XPS spectrum of Finevo-Ti was similar to that of Ti, and the peak of Ti2p was observed on Finevo-Ti (Figure 4B,D). The XPS spectra of Plasma-Ti and UV-Ti were closest to that of Tallow-Ti, but had a slight decrease in the C-H content (Figure 4B,C). As shown in Figure 4A, the atomic content of carbon on Tallow-Ti was the highest among the five groups. The atomic content of carbon on Finevo-Ti was similar to that on Ti. The atomic content of carbon on Tallow-Ti, Plasma-Ti, and UV-Ti was obviously higher than that on Ti and Finevo-Ti.

### 3.2. Cell Morphology 

**Figure 5:**
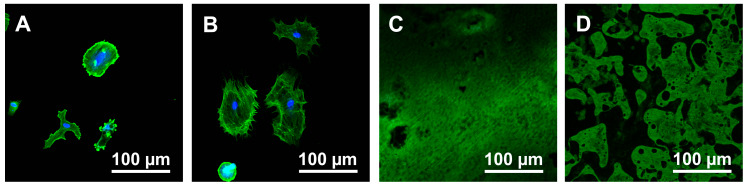
Morphological analysis of rat bone marrow mesenchymal stem cells (rBMMSCs) attached to sample disks. Tallow-Ti (**A**), Finevo-Ti (**B**), Plasma-Ti (**C**), and UV-Ti (**D**) disks were incubated with rBMMSCs for 24 h, stained with phalloidin (F-actin) and DAPI (nuclei), and visualized by fluorescence microscopy.

The morphology of cells at 24 h was observed by fluorescent staining. Compared to Tallow-Ti (Figure 5A), there were slightly more cells on Finevo-Ti (Figure 5B), and they displayed greater spreading. Some of the cells on Tallow-Ti were observed to be defective. Only flakes of oily matter were observed on Plasma-Ti and UV-Ti (Figure 5C,D). 

### 3.3. Cell Adhesion 

**Figure 6:**
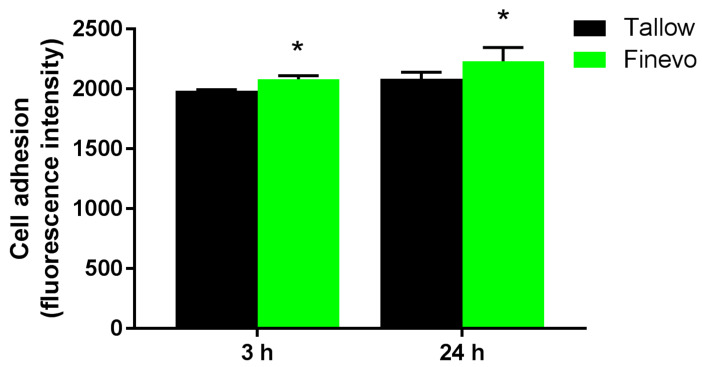
Adhesion of rat bone marrow mesenchymal stem cells on different samples after 3 and 24 h. The level of cell adhesion on Finevo-Ti after in 3 h was significantly higher than that on Tallow-Ti. This trend was also observed at 24 h. Black: Tallow-Ti, Green: Finevo-Ti, Red: Plasma-Ti, Blue: UV-Ti. (* *p* < 0.05)

Since the cells could not be observed on Plasma-Ti and UV-Ti in 24 h fluorescent staining experiment, 3 h and 24 h cell adhesion experiments were carried out only on Tallow-Ti and Finevo-Ti. The adhesion levels of rBMMSCs on the samples of Tallow-Ti and Finevo-Ti after 3 and 24 h were distinct (Figure 6). The level of cell adhesion on Finevo-Ti at 3 h was higher than that on Tallow-Ti. The same trend was observed at 24 h.

### 3.4. Alkaline Phosphatase (ALP) Activity

**Figure 7:**
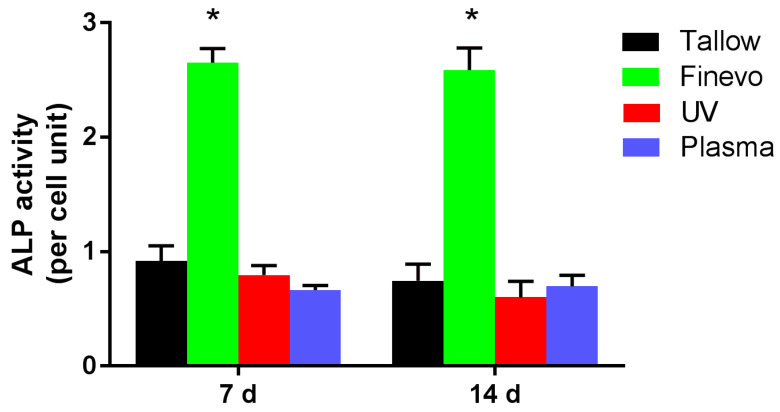
Alkaline phosphatase (ALP) activity of cells on Finevo-Ti was the highest after 7 and 14 days of differentiation. There were significant differences in ALP activity among the different samples at each time point. Cells on the Plasma-Ti and UV-Ti surfaces showed lower ALP activity than those on Tallow-Ti. Black: Tallow-Ti, Green: Finevo-Ti, Red: Plasma-Ti, Blue: UV-Ti. (* *p* < 0.05)

The ALP activity on Finevo-Ti was the highest after 7 and 14 days of differentiation (Figure 7). There were significant differences in ALP activity among the different samples at each time point. The ALP activity was found to be lower on Plasma-Ti and UV-Ti compared to that on Tallow-Ti.

### 3.5. Quantification of Calcium Deposition in the Extracellular Matrix

**Figure 8:**
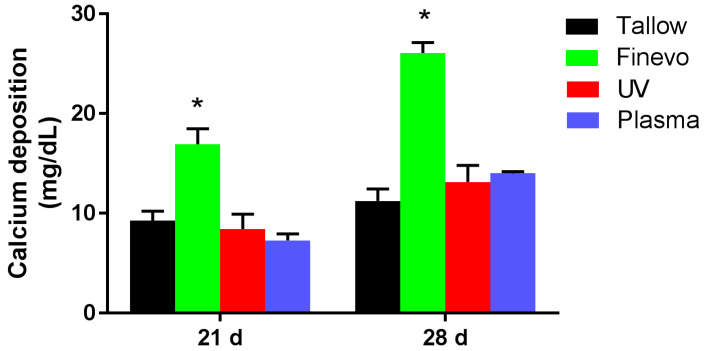
Quantification of calcium deposition, a marker for extracellular matrix mineralization, after differentiation for 21 and 28 d, in each group. Finevo-Ti showed highest calcium deposition at 21 and 28 days. The calcium deposition on Tallow-Ti, Plasma-Ti, UV-Ti at 21 and 28 days decreased as shown in the figure. Black: Tallow-Ti, Green: Finevo-Ti, Red: Plasma-Ti, Blue: UV-Ti. (* *p* < 0.05)

After differentiation for 21 and 28 days, deposition of calcium, which is a marker for extracellular matrix mineralization, on each group was quantified (Figure 8). Finevo-Ti showed the highest amount of calcium deposition at 21 and 28 days. The calcium deposition on Tallow-Ti, Plasma-Ti, and UV-Ti at 21 and 28 days was found to decrease as shown in the Figure 8.

### 3.6. Analysis of the Expression Levels of Osteogenesis-Related Genes

**Figure 9:**
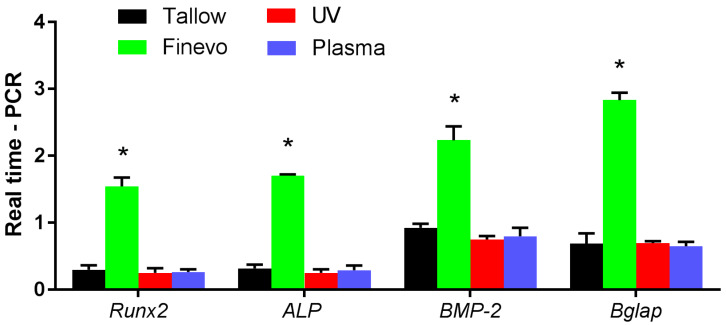
The expression level of osteogenesis-related genes evaluated by quantitative RT-PCR. The cells on Finevo-Ti showed higher mRNA levels of *ALP* and *Runx2* at 3 and 7 d, and higher mRNA levels of *BMP-2* and *Bglap* at 14 and 21 days than the cells on other groups. The mRNA levels of *ALP* and *Runx2* of Tallow-Ti, UV-Ti and Plasma-Ti at 3 and 7 days decreased in this sequence. The trend of mRNA levels of *BMP-2* and *Bglap* at 14 and 21 days in the case of Tallow-Ti, UV-Ti and Plasma-Ti was similar to that on 3 and 7 d. Black: Tallow-Ti, Green: Finevo-Ti, Red: Plasma-Ti, Blue: UV-Ti. (* *p* < 0.05)

The expression levels of osteogenesis related genes were evaluated by quantitative RT-PCR (Figure 9). The cells grown on Finevo-Ti showed higher levels of *ALP* and *Runx2* mRNAs at 3 and 7 d, and higher levels of *BMP-2* and *Bglap* mRNAs at 14 and 21 d relative to the levels in the cells on the other samples. The mRNA levels of *ALP* and *Runx2* in the cells on Tallow-Ti, UV-Ti, and Plasma-Ti at 3 and 7 d decreased in this sequence. The *BMP-2* and *Bglap* mRNA levels at 14 and 21 d in the cells on Tallow-Ti, UV-Ti and Plasma-Ti were similar to the respective levels at 3 and 7 d.

## 4. Discussion

Whether the contamination is of hydrocarbons adsorbed from the air during storage or of oil and other residues remaining after the processing, it affects the surface of the implant [28,29,30]. It has been pointed out that the surface of Ti implants currently used in clinics is contaminated with hydrocarbons [13,14,15,16,31,32]. The changes in the surface properties of Ti implants affect the attachment, proliferation, and differentiation of osteoblasts, which eventually affect the osseointegration after implantation and the success rate of implantation [33]. There is evidence that hydrocarbons have an inhibitory effect on the growth and differentiation of osteoblastic cells [12]. Therefore, it is particularly important to effectively remove the hydrocarbon contamination from the surface of Ti implants so as to promote the osteointegration of osteoblasts, improve the success rate of implantation, and prevent the occurrence of peri-implantitis. The tallow used in this experiment is an organic compound, which effectively mimics the carbon contamination on the surface of Ti implants. Studies have shown that UV [18] or plasma [19,20,21] treatment can remove hydrocarbon contamination from the surface of Ti implants to a certain extent and these methods improve the surface bioactivity of the Ti implants. There is an ultrasonic treatment method using Finevo antibacterial solution that can effectively remove the contamination on the surface of the abutment acquired during the processing, but its effect in cleaning the surface of the implant is not clear [23,24]. Therefore, in this study, we compared the above three methods of cleaning the tallow contamination from the surface of Ti disks.

The results presented herein confirm our previous hypothesis. Of the three methods, the Finevo cleaning system had the best effect in removing tallow from the Ti disks. Moreover, in vitro cell attachment and osteogenic activity on Finevo-Ti were obviously higher than on Tallow-Ti, Plasma-Ti, and UV-Ti.

Titanium is a metal with extremely high biological activity; it is covered with an oxide layer (TiO_2_) on the surface and is often used to make dental implants. Theoretically, the amount of Ti on the surface can be up to 30%, but in most cases, the amount is reduced because of the adsorption of hydrocarbons. Therefore, it has been suggested that it is reasonable to use a normal clean Ti implant surface containing 18% Ti [34]. During the manufacturing process of the implants, because of contact with the organic lubricating oil, a large number of hydrocarbons are easily attached on the surface of Ti, thereby, increasing the carbon content and reducing the property of Ti [30]. The results of surface analysis proved that tallow was visible on the surface of the Ti disks soaked with tallow and the original structure of the surface of the Ti disks could not be observed. This proves that tallow was successfully attached to the surface of the Ti disks. Compared with the plasma and UV treatment methods, the Finevo cleaning system has a stronger effect in removing the tallow. Moreover, consistent results were also observed for XPS analysis. The accumulation of hydrocarbons on the Ti surface is accompanied by a significant increase in C1s and a decrease in Ti2p and O1s. The decrease in the amount of C-H indicates a decrease in the amount of hydrocarbons on the surface of the material. As predicted by us, the Finevo cleaning system showed a relatively strong ability to remove C-H, and the surface properties of Finevo-Ti were found to be the closest to that of Ti, with a carbon content of 19%. Although the C-H content on the Plasma-Ti surface was slightly lower than that on Tallow-Ti, it was still much higher than that on the Ti surface, and the C-H content on the UV-Ti surface was about the same as on Tallow-Ti. Moreover, although only a small amount of the tallow on the surface of Plasma-Ti and UV-Ti was removed, the peak of Ti2p was not detected. Therefore, it can be considered that, compared with the plasma and UV treatment, the Finevo cleaning system can recover the surface structure of Ti disks contaminated with organic compound to a greater extent. On the other hand, the ability of the plasma and ultraviolet treatments to clean the tallow was limited, because the thick organic layer could not be penetrated in these treatments.

It is obvious that in vitro, the cells on Finevo-Ti showed better osteogenic activity than those on the other groups. First of all, Finevo-Ti showed the highest cell adhesion at 6 and 24 h. The result of fluorescence staining showed that at 24 h, the cells adhering to Tallow-Ti surface were less than those on Finevo-Ti, and some cells were defective; the cells on Finevo-Ti relatively fully covered the surface, whereas a large amount of tallow could be observed on the surfaces of Plasma-Ti and UV-Ti, but no cells could be seen. Based on these results, it can be proved that the existence of a large amount of tallow on the Ti surface can hinder the adhesion of cells to the surface. Finevo cleaning system can effectively clean the large amount of tallow attached to the surface. Some studies have pointed out that the surface wettability of materials may decrease with the adsorption of organic compounds [12]. The improvement in the wettability can promote the adsorption of cell adhesion proteins and cell adhesion [35]. We observed the difference in the hydrophilicity among the different groups of samples, and the hydrophilicity of Tallow-Ti, UV-Ti, and Plasma-Ti was observed to decrease significantly, perhaps due to the attachment of a large amount of tallow. However, the specific reactions and changes in the tallow or titanium disks in the case of the three treatments need to be studied further to explain the obvious differences among the different groups. Studies have shown that initial attachment of cells has a very important effect on the normal functioning of cells and subsequent tissue integration, specifically affecting the ability of cells to proliferate, grow, and differentiate [36,37,38]. Therefore, the removal of carbon contamination from Ti implants plays an important role in promoting the adhesion and growth of cells on the implants at the initial stage of implantation.

The osteogenic differentiation can be divided into three stages: proliferation, matrix maturation, and mineralization [39]. When the cells gradually transit from the proliferative phase to the matrix maturation stage, the expression of ALP activity-related genes is significantly enhanced. In this study, the cells on Finevo-Ti expressed higher ALP activity per unit cell at 7 and 14 days, which proved that the presence of organic compounds, such as tallow, can affect the osteogenic differentiation of cells, whereas the removal of tallow by the Finevo cleaning system effectively improved the living environment of the cells and promoted the interaction among cells, thereby, promoting osteogenic differentiation at an early stage [40,41]. As a marker of terminal osteogenic differentiation, calcium deposition in the extracellular matrix reached the maximum at the mineralization stage; this plays an important role in evaluating the efficiency of osteogenic differentiation of cells [42]. The calcium deposition by the cells in the four groups (Tallow-Ti, Finevo-Ti, Plasma-Ti, and UV-Ti) was high on days 21 and 28. We speculated that the clearance of tallow in the Finevo-Ti group could effectively improve the early attachment and expansion of cells, promote cell–cell interaction, and thus, promote osteogenic differentiation. On the contrary, because the attachment of tallow seriously affected the cell growth, a large number of cells died and cell fragments were deposited, because of which the results for the other three groups were on the higher side. Further research is needed for clarity in this regard.

ALP and Runx2 are key transcription factors expressed during the early stages of osteogenic differentiation, whereas BMP and Bglap are important factors in the late stage of osteogenic differentiation [27,43,44]. The significant difference in the expression of osteogenesis-related genes on Tallow-Ti and Finevo-Ti showed the inhibitory effect of tallow on osteogenic differentiation and the efficient decontamination ability of the Finevo cleaning system. However, the relatively limited decontamination ability of the plasma and UV treatments was not sufficient to remove a large amount of tallow contamination from the Ti surface, and could not restore the original bioactivity of Ti.

It has been found that the surface of Ti implants currently used in clinics is contaminated with hydrocarbons [12,13,14,15,29,30]. However, little attention has been paid to the methods for the cleaning of Ti implants before implantation. Presently, a set of best cleaning processes for cleaning the surface contamination of Ti implants has not been developed. Consistent with the results of previous studies, this study shows that a large number of carbons attached to the surface of Ti can significantly inhibit cell attachment and osteogenic differentiation [11,12,45]. The comparison of the three cleaning methods showed that the efficacy of the Finevo cleaning system on the removal of carbons attached to the surface of Ti was significantly higher than that of the plasma and UV treatment methods. More in-depth research is needed to identify the mechanisms underlying the decontamination and restoration of the bioactivity of Ti using the three methods described herein. The removal of carbons from the surface of Ti implants may be an important step in promoting the bioactivity and osseointegration of Ti. Therefore, more studies are needed on the cleaning methods, such as the Finevo cleaning system, and for development of an effective cleaning process that promotes the osseointegration of Ti implants and improves the success rate of implantation.

## 5. Conclusions

Surface studies presented here show that the Finevo cleaning system can effectively remove the tallow contamination on the surface of Ti disks and can restore the original surface morphology. In vitro experiments showed that the effective tallow decontamination ability of the Finevo cleaning system successfully improved the bioactivity and osteogenic differentiation ability of contaminated Ti disks. Therefore, the Finevo cleaning system has the potential to clean the implant surface in clinics and is worthy of further in-depth study.
